# Comparison of microscopic and metagenomic approaches to identify cereal pathogens and track fungal spore release in the field

**DOI:** 10.3389/fpls.2022.1039090

**Published:** 2022-10-20

**Authors:** Paola Pilo, Colleen Lawless, Anna M. M. Tiley, Sujit J. Karki, James I. Burke, Angela Feechan

**Affiliations:** ^1^ School of Agriculture & Food Science and UCD Earth Institute, University College Dublin, Belfield, Ireland; ^2^ School of Biology and Environmental Science and UCD Earth Institute, University College Dublin, Belfield, Ireland; ^3^ Department of Agriculture, Food and the Marine, Celbridge, Ireland; ^4^ Institute for Life and Earth Sciences, School of Energy, Geosciences, Infrastructure and Society, Heriot-Watt University, Edinburgh, United Kingdom

**Keywords:** Spore trap, ascospore, microscopy, metagenomic, fungal pathogen, *Zymoseptoria tritici*, *Fusarium* spp., wheat

## Abstract

Wheat is one of the main staple food crops, and 775 million tonnes of wheat were produced worldwide in 2022. Fungal diseases such as Fusarium head blight, Septoria tritici blotch, spot blotch, tan spot, stripe rust, leaf rust, and powdery mildew cause serious yield losses in wheat and can impact quality. We aimed to investigate the incidence of spores from major fungal pathogens of cereals in the field by comparing microscopic and metagenomic based approaches for spore identification. Spore traps were set up in four geographically distinct UK wheat fields (Carnoustie, Angus; Bishop Burton, Yorkshire; Swindon, Wiltshire; and Lenham, Kent). Six major cereal fungal pathogen genera (*Alternaria* spp.*, Blumeria graminis, Cladosporium* spp., *Fusarium* spp., *Puccinia* spp., and *Zymoseptoria* spp.) were found using these techniques at all sites. Using metagenomic and BLAST analysis, 150 cereal pathogen species (33 different genera) were recorded on the spore trap tapes. The metagenomic BLAST analysis showed a higher accuracy in terms of species-specific identification than the taxonomic tool software Kraken2 or microscopic analysis. Microscopic data from the spore traps was subsequently correlated with weather data to examine the conditions which promote ascospore release of *Fusarium* spp. and *Zymoseptoria* spp. This revealed that *Zymoseptoria* spp. and *Fusarium* spp. ascospore release show a positive correlation with relative humidity (%RH). Whereas air temperature (°C) negatively affects *Zymoseptoria* spp. ascospore release.

## Introduction

Every year approximately 15 – 20% of wheat yields are lost due to fungal pathogens (FAO, 2022; Figueroa et al., 2018). Yield losses in the UK due to Septoria tritici blotch (STB) caused by *Zymoseptoria tritici* are around 20% on average (Fones and Gurr, 2015). In addition to yield loss Fusarium head blight (FHB) also contaminates grain with deoxynivalenol that is harmful to human and animal health (Rocha et al., 2005). One of the ways which cereal fungal pathogens can spread to new hosts is *via* spore release into the atmosphere from infected plants or stubble (Eversmeyer and Kramer, 1987; Suffert et al., 2011).

Aerobiology studies applied to plant pathology have focused on fungal spore dispersal in the air by using passive or active spore traps as a sampling method (Calderon et al., 1995; Cordo et al., 2017; Aguayo et al., 2018; Schiro et al., 2018; Woo et al., 2018). Passive spore traps include filter papers or microscope slides coated with an adhesive matrix, mounted onto a support. The spores in the air naturally land on the sampling surface by gravity or wind (Cordo et al., 2017; Aguayo et al., 2018; Schiro et al., 2018; Woo et al., 2018). In contrast, active spore traps use a mechanical and electrical system to draw a constant volume of air inside the mechanism. This allows spores to adhere to adhesive tape, a microscope slide, a micro tube, or a filter, depending on the type of machine in use (Calderon et al., 1995; Grinn-Gofroń and Rapiejko, 2009; Jackson and Bayliss, 2011)

The analysis following spore trap sampling ranges from more “traditional” approaches such as microscopy for spore counting and identification (Calderon et al., 1995; Cordo et al., 2017; Schiro et al., 2018) to molecular techniques such as Polymerase Chain Reaction (PCR) and New Generation Sequencing (NGS), often involving analysis of the ribosomal internal transcribed space (ITS) region (Woo et al., 2018; Aguayo et al., 2018). Metagenomics utilises NGS to analyse the genomes of all the organisms from an environmental sample. This type of environmental sampling in the field can allow the detection of a wide range of crop and plant pathogens since no prior knowledge of the pathogens present is required (Yang et al., 2022). Microscopy of airborne spores is a well-established technique and, despite being time consuming, provides detailed information. For example, timing of spore release can be correlated with external factors such as the time of day (dawn and dusk) (Calderon et al., 1995) and changes in weather conditions (Cordo et al., 2017; Schiro et al., 2018).

PCR based methods have also been used for correlation with weather data where gene copy numbers obtained by qPCR analysis were statistically compared with average weather data (Choudhury et al., 2016). Molecular based approaches for the study of airborne spores can be very useful in terms of identification at the species level. Previous research by Woo et al. (2018) used weekly DNA extractions, and sequencing of the ITS region amplicons to determine which species were present in the atmosphere between two different collection conditions (dry and wet). Whereas, Aguayo et al. (2018) assessed high-throughput sequencing with metabarcoding to compare different types of passive spore trap samples and DNA extraction methods.

In this study the presence of airborne spores from fungal pathogens of cereals were assessed and compared using microscopy and two metagenomic based methods (BLAST based and Kraken2) (Wood et al., 2019). Examples of cereal pathogens identified include *Alternaria* spp.*, Cladosporium* spp.*, Blumeria graminis*, *Fusarium* spp.*, Puccinia* spp., and *Zymoseptoria tritici*. The samples were collected from volumetric spore traps over four UK wheat field sites (Carnoustie, Angus; Bishop Burton, Yorkshire; Swindon, Wiltshire and Lenham, Kent). Weather data such as air temperature (°C), rainfall (mm), relative humidity (%RH), and wind speed (Km/h) were correlated with bi–hourly time points of spore release for the Ascomycete fungal pathogens of wheat *Zymoseptoria* spp. and *Fusarium* spp., using microscopy. This provided an overview of the fungal pathogens present at the different field sites and revealed that spore release is positively correlated with relative humidity (%RH) but negatively correlated with air temperature (°C).

## Methods

### Spore trap set-up

**Figure 1:**
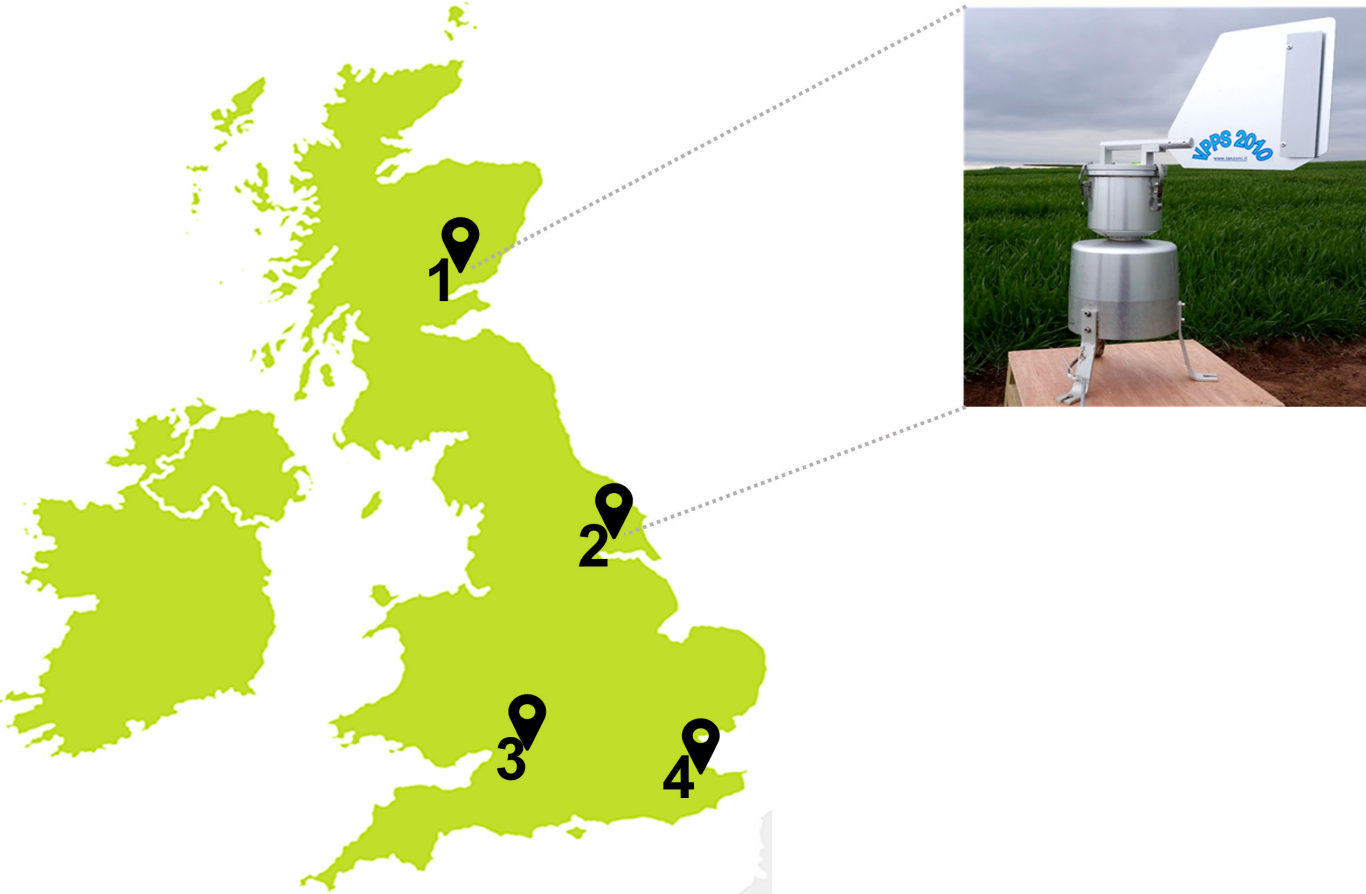
UK spore traps locations at wheat fields in: **(1)** Carnoustie, Angus (North-East); **(2)** Bishop Burton, Yorkshire (Central-East); **(3)** Swindon, Wiltshire (South-West); and **(4)** Lenham, Kent (South-East). Volumetric Pollen and Particle Sampler, VPPS^®^ 2010 by LANZONI srl in Carnoustie and Bishop Burton; Seven-Day Recording Volumetric Spore Trap by ^©^Burkard Manufacturing Co Ltd in Swindon and Lenham.

Spore traps were installed at each of the four UK sites 1) Carnoustie, Angus; 2) Bishop Burton, Yorkshire; 3) Swindon, Wiltshire; and 4) Lenham, Kent (**Figure 1**). A volumetric Pollen and Particle Sampler (VPPS^®^ 2010, by LANZONI srl) was installed at the Carnoustie and Bishop Burton field sites (**Figure 1A**). A Seven-Day Recording Volumetric Spore Trap (^©^Burkard Manufacturing Co Ltd) was installed at the Swindon and Lenham field sites (**Figure 1B**). Both type of spore traps record volumetric samples for seven days. Each machine is equipped with a rotating drum where a SILKOSTRIP (Lanzoni), a polyester pre–siliconed sampling tape, is mounted for each week of collection. A clockwork mechanism rotates the drum clockwise while air enters through the orifice allowing spores to be deposited on the tape, with a progression of 2 mm every 2 hours (h). Each spore trap is powered by a 12–Volt battery. The flow rate is checked after installation with a flowmeter provided by the manufacturer, as spore traps operate with an airflow of 10 *Lmin^-^* ^1^. The spore traps were set up in each of the four UK wheat fields, and air samples were collected consecutively for seven days, every two weeks, from 4^th^ May 2018 until 17^th^ of August 2018.

### Spore trap tape preparation

**Figure 2:**
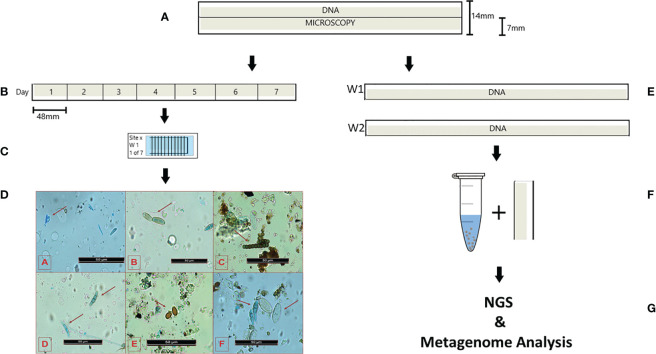
Schematic summarizing the preparation of the spore trap tapes for microscopy and metagenomic analysis. **(A)** the full tape was split longitudinally and divided ½ for microscopy and ½ for DNA extraction; **(B)** the microscopy half was divided into 7 sections (48 mm wide), each representing one day of collection; **(C)** tape sections were stained with solution of 10% Trypan-blue-lactophenol 90% colorless glycerin, and mounted on microscope slides, which were segmented in 12 parts 2mm wide. Each 2mm section represents 2 hours of collection; **(D)** Representative images of different fungal spores from the spore trap tape microscope slides. Red arrows indicate: [A] *Zymoseptoria* spp. ascospores, [B] *Cladosporium* spp., [C] *Torula herbarum* [D] *Fusarium* spp. Ascospores, [E] *Puccinia* spp. [F] *Alternaria* spp. (left), *Blumeria graminis* (right); **(E)** Two half tapes from two consecutive weeks of collection were used for monthly DNA extraction; **(F)** tape sections were used for DNA extraction using the Qiagen DNeasy Soil Kit and 400 – 600 µm acid – washed beads; **(G)** DNA extracted was sent for NGS and DNA reads analyzed for metagenomic of fungal pathogens.

Following collection, insects and large debris were removed and trap tapes were stored at 4°C prior to preparation. Each of the spore trap tapes were divided into two longitudinal halves; the first half of the tape was used for downstream microscopy and the second half used for DNA extraction and metagenomic analysis (**Figure 2A**). A total of eight half tapes corresponding to eight weeks (two weeks each from the months of May, June, July, and August) from 2018, were used to perform microscopy analysis, and a total of six half tapes corresponding to six weeks (two weeks from the months of June, July, and August) were used for metagenomic analysis for all sites (**Figure 2**).

### Microscopy

For microscopic analysis, each of the half tapes were divided into seven segments, each measuring 48 x 7 mm (L X W) and representing each of the seven days of collection (**Figure 2B**). Individual segments were then placed on a microscope slide. A solution of 10% Trypan – blue – lactophenol (Boedijn, 1956) and 90% colorless glycerin (100%) (~300µl *per* slide) was evenly distributed over the tape to stain the fungal spores. The tapes were then covered with a glass slide and left to dry. Once dry, a marker pen was used to divide the slides into 12 x 2 mm transverse sections, each one corresponding to 2-hour (2 h) time points, so that one slide represents 24 hours of sampling (**Figure 2C**).

Spore sampling was undertaken over two weeks per month over four months (MayA-August) in 2018. Therefore, two spore trap tapes were collected per month per site. A total of 14 slides (one for each day of the two weeks) were analyzed *per* month *per* site. For each site, there was therefore a total of 56 slides, representing a total of 56 days.

Microscopy was performed using a Leica DM5500B microscope equipped with a Leica DFC 310 FX Camera. Fungal taxa were identified *via* visual assessment based on spore morphology and appearance (**Figure 2D**) (Lacey and West, 2007). Spores were counted for three random fields of view (1 field = 321 x 240 µm) *per* each of the 12 x 2 mm transverse sections with X40 magnification. The number and type of spores available was recorded. The fungal spores were adjusted to the concentration of spores per cubic meter of air using the following formula:


$$\text{N }\times \text{CF}=\text{N}\times \frac{0.28}{width\;of\;one\;traverse\;\left(mm\right)}$$

**Figure 3:**
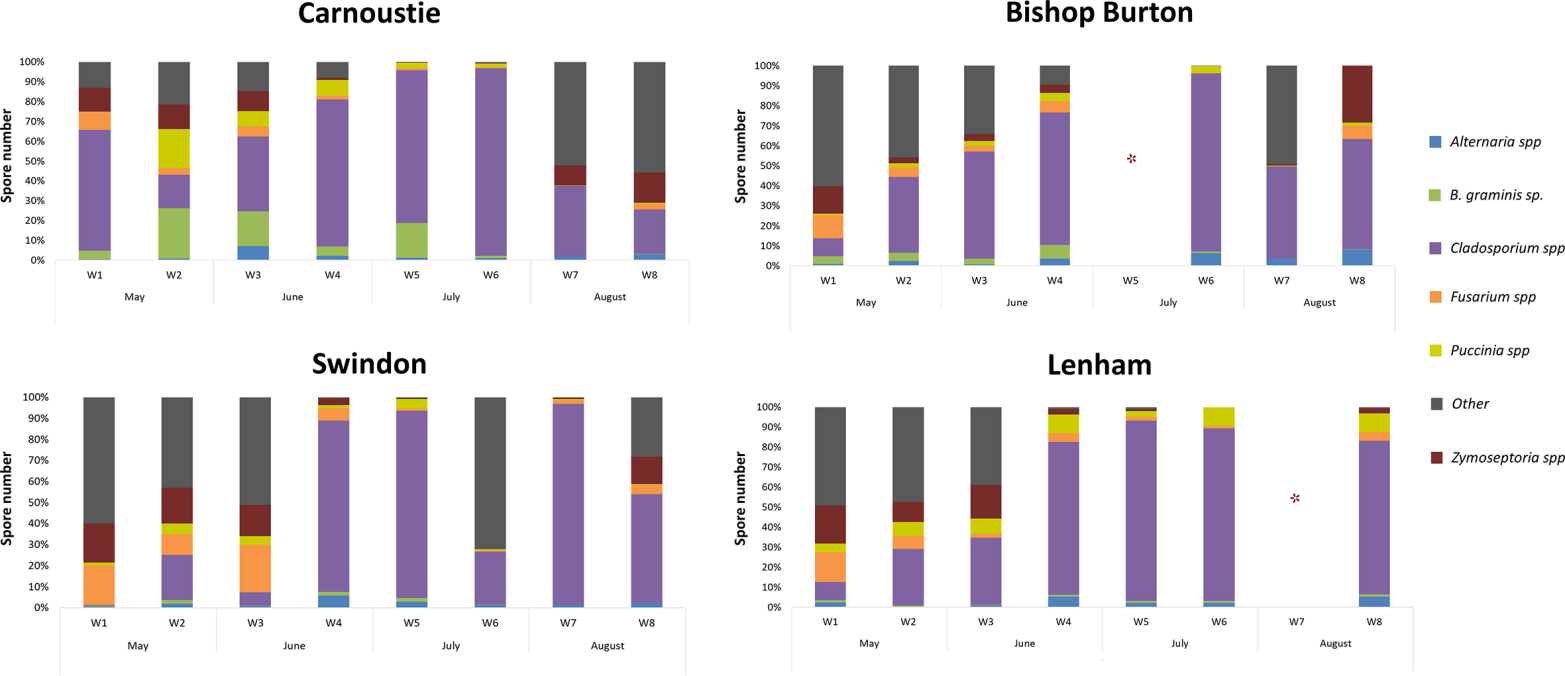
Weekly percentages of spores from the six major pathogens recognized and detected with the microscopic analysis at the four sites. Asterisk (*) denotes missing data in week 5 from the Bishop Burton field site and at week 7 at the Lenham field site due to mechanical failure of the spore trap.

Where (N) is the daily total number of spores, multiplied by the Correction Factor (CF) where the combination of microscope/lens, used will give the daily mean concentration of fungal spores per cubic meter (Lacey and West, 2007). In this case a Leica DM5500B microscope with a DFC 310 FX Camera, and X40 lens magnification corresponds to a width of 321 µm (0.321 mm). The results from each day of sampling were summed to obtain a weekly count of fungal spores per cubic meter (**Figure 3**). Percentages of each spore type were calculated weekly *per* site (**Table 1**).

**Table 1:** Microscopy results displayed as weekly, week 1 (W1) to week 8 (W8) percentage of fungal cereal pathogen abundances.

Site	*Cereal Fungal Pathogen*	W1	W2	W3	W4	W5	W6	W7	W8
**Car**	*Alternaria* spp.	0.41%	0.80%	7.13%	2.20%	1.21%	0.97%	1.81%	3.09%
**BB**	*Alternaria* spp.	0.77%	2.39%	0.64%	3.63%		6.28%	3.65%	8.01%
**SW**	*Alternaria* spp.	0.58%	1.95%	0.73%	5.83%	2.91%	1.26%	1.23%	2.06%
**Len**	*Alternaria* spp.	2.28%	0.22%	0.99%	5.23%	2.14%	2.13%		5.26%
**Car**	*Blumeria graminis*	4.31%	25.46%	17.48%	4.67%	17.57%	1.24%	0.04%	0.14%
**BB**	*Blumeria graminis*	3.94%	4.13%	3.01%	6.85%		0.80%	0.02%	0.34%
**SW**	*Blumeria graminis.*	0.32%	1.65%	0.10%	1.68%	1.58%	0.14%	0.06%	0.05%
**Len**	*Blumeria graminis*	1.16%	0.61%	0.14%	1.09%	0.99%	1.06%		1.10%
**Car**	*Cladosporium* spp.	61.04%	16.95%	37.70%	74.08%	77.08%	94.51%	35.79%	22.47%
**BB**	*Cladosporium* spp.	9.09%	37.87%	53.47%	66.22%		89.05%	45.95%	55.05%
**SW**	*Cladosporium* spp.	0.32%	21.42%	6.53%	81.46%	89.13%	25.24%	95.62%	51.78%
**Len**	*Cladosporium* spp.	9.13%	28.37%	33.49%	76.34%	90.15%	86.17%		76.75%
**Car**	*Fusarium* spp.	9.17%	3.21%	5.17%	2.08%	0.95%	0.48%	0.14%	2.64%
**BB**	*Fusarium* spp.	11.37%	4.68%	2.79%	5.65%		0.12%	0.12%	6.50%
**SW**	*Fusarium* spp.	18.82%	9.88%	22.47%	5.65%	0.89%	0.58%	1.84%	4.51%
**Len**	*Fusarium* spp.	14.80%	6.54%	1.95%	4.37%	1.75%	1.06%		4.39%
**Car**	*Puccinia* spp.	0.00%	19.68%	7.76%	7.87%	2.64%	1.83%	0.13%	0.64%
**BB**	*Puccinia* spp.	0.88%	2.18%	2.36%	4.02%		3.53%	0.39%	1.56%
**SW**	*Puccinia* spp.	1.36%	5.06%	4.21%	1.73%	4.62%	0.49%	0.50%	0.25%
**Len**	*Puccinia* spp.	4.45%	6.89%	7.65%	9.30%	3.03%	9.57%		9.35%
**Car**	*Zymoseptoria* spp.	12.06%	12.45%	9.87%	1.01%	0.18%	0.38%	9.85%	15.35%
**BB**	*Zymoseptoria* spp.	13.70%	2.94%	3.62%	4.04%		0.06%	0.46%	28.53%
**SW**	*Zymoseptoria* spp.	18.49%	16.96%	14.75%	3.11%	0.34%	0.01%	0.47%	13.14%
**Len**	*Zymoseptoria* spp.	18.95%	9.87%	16.82%	2.54%	0.80%	0.00%		2.55%
**Car**	Other	13.02%	21.45%	14.89%	8.10%	0.37%	0.59%	52.25%	55.67%
**BB**	Other	60.25%	45.81%	34.10%	9.58%		0.15%	49.42%	0.00%
**SW**	Other	60.09%	43.08%	51.21%	0.54%	0.53%	72.28%	0.30%	28.22%
**Len**	Other	49.23%	47.50%	38.95%	1.12%	1.13%	0.00%		0.59%

Same genera/species clustered together for the 4 sites (Car = Carnoustie; BB = Bishop Burton; Sw = Swindon; Len = Lenham). The color scheme in each of the names follow the same color distribution of **Figure 3** for comparison.

Microscopic results for spores from the ascomycete fungal pathogens, *Zymoseptoria* spp. and *Fusarium* spp., from all four sites were analyzed based on 2 h time points. The spore release data were correlated to weather data, specifically air temperature (°C), rainfall (mm), relative humidity (%RH), and wind speed (Km/h), to elucidate whether there was a correlation between spore release and weather conditions. The weather data was provided by Origin Enterprises plc. Weather stations were located at each of the four sites and data was collected every 15 minutes.

### DNA extraction and processing

DNA extraction for Whole Genome Sequencing (WGS), was performed using a Qiagen DNeasy Soil Kit with the addition of 400–600 µm acid washed beads. Two half – tapes from the two weeks of collection for each month were pooled to generate a monthly sample from June to August for all four sites (**Figure 2E**). This therefore resulted in a total of 12 DNA samples per site. DNA extraction from May samples was unsuccessful.

Each tape was cut into 7 mm x 40 mm (W x L) sections and placed into a 2 ml screw – lid tube provided with the DNeasy Soil Kit, in which 300mg of 400–600 µm acid – washed beads were added. Each tube containing the tape was vortexed for five minutes, the processed tape was then exchanged for a new piece and vortexing was repeated for each month.

Once all tape segments were processed, the DNeasy Soil Kit protocol was followed using the manufacturer’s instructions (**Figure 2F**). WGS was performed by Eurofins Genomics using a pipeline for low yield samples (DNA yield< 10 ng/µl). A metagenomic library was constructed, using Illumina^®^ sequencing technology with 2 x 150 bp reads and with a minimum of 5 million reads per sample.

### Metagenomics

DNA reads were analyzed using the bioinformatic OmicsBox Software by Bioinformatics and Valencia, 2019 (**Figure 2G**). Quality control of the reads and pre–processing was done using FASTQ. Metagenomic assembly of the reads was performed by MEGAHIT (Li et al., 2015). Gene and protein prediction from the assembled contigs was performed with FragGeneScan (Rho et al., 2010). Once obtained, protein prediction FASTA files were used for a Basic Local Alignment Search Tool (BLAST – p) search against non-redundant protein database using the OmicsBox online cloud. BLAST Species Hits from the predicted protein sequences were retrieved, characterized, and classified for species recognition based on the first five highest hits with an e – value ≤ 0.01 and Sim mean ≥ 60%. BLAST results were then analyzed with MEGAN Community Edition (version 6.21.10, built 29 Jul 2021) (Huson et al., 2007; Huson et al., 2016) to determine taxonomic relative abundances, in each site *per* each month.

As a control method for the BLAST analysis, the taxonomic classification tool Kraken2 was used, from OmicsBox. Kraken2 is a taxonomic classification system using exact *k – mer* matches to achieve high accuracy and fast classification speeds. This classifier matches each *k – mer* within a query sequence to the lowest common ancestor (LCA) of all genomes containing the given *k – mer* (Wood et al., 2019). This analysis was performed using the raw cleaned FASTQ reads. To compare both taxonomic systems, species diversity indexes (Shannon – Wiener and Simpson’s) were calculated for both methods (Peet, 1975).

### Statistical analysis

Statistical analysis for the comparison of results from both metagenomic methods were performed on results from BLAST and Kraken2. Species diversity indexes Shannon-Wiener and Simpson’s were calculated for both methods (Peet, 1975). A two – way ANOVA test was performed using GraphPad Prism version 8.0.0 for Windows, GraphPad Software, San Diego, California USA (www.graphpad.com), to determine the difference between the Indexes means for the two methods.

Correlation between weather data from the four sites and M spore release data at two hour time points were analyzed using Prism – GraphPad (www.graphpad.com), and IBM SPSS Statistics 26 (IBM-Corp, 2019). The non – parametric Spearman’s rho correlation coefficient (Spearman's Rank, 2008) was used to assess *Zymoseptoria* spp. and *Fusarium* spp. ascospore release against the air temperature (°C), rainfall (mm), relative humidity (%RH), and wind speed (Km/h).

## Results

### Abundance of fungal cereal pathogen spores across UK wheat field sites using microscopy

Approximately 20 taxa were identified using microscopy (*Alternaria spp., Aspergillus spp., Blumeria graminis, Cladosporium spp., Claviceps purpurea, Cochliobolus/Bipolaris spp., Epicoccum spp., Fusarium spp., Gaeumannomyces tritici, Helicomyces spp., Myxomycetes, Penicillium spp., Pithomycetes, Pleospores, Puccinia spp., Tilletiopsis spp., Torula spp., Trichotechium roseum, Zymoseptoria spp.*). Microscopy classification of spores in the *Fusarium* or *Alternaria*, *Cladosporium*, and *Puccinia* genus were counted as a unit at the genus level. However, microscopy was impacted by the malfunction of the spore traps at Bishop Burton in week five (July) and Lenham week seven (August) and as a result only DNA extraction was possible for these weeks.

Microscopic analysis revealed a broad diversity in the fungal species present on the spore trap tapes. Of these, cereal pathogens belonging to six genera (*Alternaria* spp.*, Blumeria graminis, Cladosporium* spp.*, Fusarium* spp.*, Puccinia* spp. and *Zymoseptoria* spp.*)* were consistently identified throughout the sampling season at every site and these were selected for downstream spore counting. The remaining fungal spores were recorded as “*Other*”. The percentage of spore abundance for each fungal cereal pathogen genera was recorded per site *per* week (**Figure 3**). The percentage of the total amount of spores *per* sample by pathogen genera are shown in **Table 1**.

A high percentage of *Blumeria graminis* conidia were identified at the Carnoustie field site followed by the Bishop Burton field site throughout the sampling season (**Figure 3**). Carnoustie had the highest percentage of *B. graminis* conidia, with 25.46% in week 2 (May), 17.48% in week 3 (June) and 17.47% in week 5 (July). The percentage of *B. graminis* conidia decreased at the Carnoustie and Bishop Burton sites towards the end of the season in week 6 (July), week 7 and week 8 (August), where values registered were between 0.02% and 1.24% (**Table 1**). Low percentage levels of *B. graminis* were identified at the Swindon and Lenham field sites throughout the sampling season. For example, at Swindon, the percentage of *B. graminis* was highest in week 4 (June) 1.68% and lowest in week 8 (August) 0.05%. At Lenham, the percentage of *B. graminis* was highest in week 1 (May) at 1.16% and lowest in week 3 (June) 0.14% (**Figure 3**, **Table 1**).

The presence of *Zymoseptoria* spp. ascospores was detected throughout the season at all four sites. In week 1 (May) there was a high percentage of *Zymoseptoria* spp. at all sites (Carnoustie 12.06%, Bishop Burton 13.70%, Swindon and Lenham >18%). Presence of *Zymoseptoria* spp. ascospores decreased at the Bishop Burton site to 2.94% in week 2 (May) and to 3.62% in week 3 (June). The abundance *of Zymoseptoria* spp. ascospores at the remaining sites were between ~10% to ~17% (**Figure 3**, **Table 1**). A decrease in the percentage of *Zymoseptoria* spp. ascospores at all sites from week 4 (June) to week 7 (August) was recorded. This was with the exception of Carnoustie, where there was an increase from ~0.4% in week 6 (July) to ~10% in week 7 (August) (**Table 1**). The percentage of *Zymoseptoria* spp. ascospores increased at the end of August (week 8) at all sites. The highest percentage of *Zymoseptoria* spp. ascospores was registered at week 8 in Bishop Burton at 28.53% and the lowest percentage was registered at week 8 in Lenham at 2.55% (**Table 1**, **Figure 3**).

*Fusarium* spp. ascospore release followed a similar pattern to *Zymoseptoria* spp. ascospore release (**Figure 3**). Higher ascospore percentages were recorded at the beginning of the sampling season in week 1, from 9.17% in Carnoustie to 18.82% in Swindon (**Table 1**). This followed a decrease by the end of June (week 4) in all the sites, with percentages of *Fusarium* spp. between 2.08% and 5.65% (**Table 1**). Percentages of *Fusarium* spp. ascospores remained low across the four sites in weeks 6 and 7 (0.12% ≤ X ≤ 1.84%). A slight increase was recorded by the end of August (week 8) with percentages between 2.64% and 6.50% (**Table 1**).

Monthly results from microscopic analysis for the whole season demonstrated that spores belonging to the *Cladosporium* spp. genus were the most abundant spore type at all four sites. The maximum percentage of *Cladosporium* spp. spores were registered at Carnoustie in July (90.72%) and the lowest percentage were registered at Swindon in May (11.19%) (**Table S1**). *Zymoseptoria* spp. was the second most abundant single pathogen over the four sites. The highest percentage presence of *Zymoseptoria* spp. ascospores was recorded at Swindon in May (17.70%) and the lowest percentage presence was observed at Bishop Burton in July (0.06%). Generally, the month of May registered the highest percentage of *Zymoseptoria* spp. ascospores across all sites (**Table S1**). The monthly percentages of *Fusarium* spp. ascospores also showed a higher percentage presence in May for all sites (6.66% in Carnoustie, 9.63% in Bishop Burton, 14.21% in Swindon, and 10.21% in Lenham). However, the highest presence of *Fusarium* spp. ascospores was found in Swindon in June (20.93%) (**Table S1**). While the lowest percentage of *Fusarium* spp. was found in July over the four sites (0.58% at Carnoustie, 0.12% in Bishop Burton, 0.68% in Swindon, and 1.74% in Lenham) (**Table S1**).

### Correlation between weather conditions and *Zymoseptoria* spp. and *Fusarium* spp. spore release using microscopy

**Figure 4:**
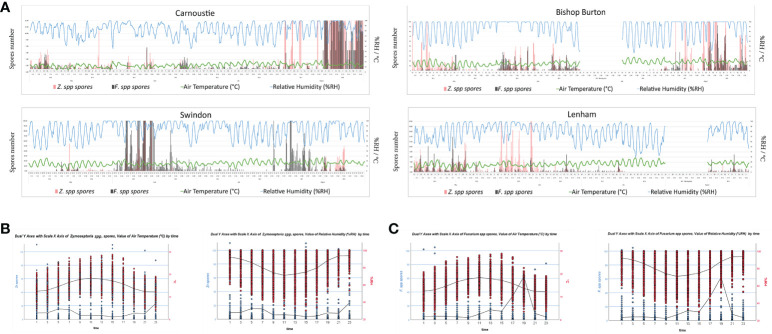
**(A)** Temporal distribution of *Z.* spp. (*Zt*, Pink bars) and *Fusarium* spp. (*F.*spp. Grey bars) ascospore release, plotted on the primary Y axis, against air temperature (Green line) and relative humidity (Blue line), plotted on the secondary Y axis; for all the four sites. **(B)** Dual Y axis with scale X axis of *Zymoseptoria* spp. or *Fusarium* spp. ascospores and value of air temperature by 2h timepoints. Values for all the 4 sites were used to generate the negative correlation curve. Graphs with IBM SPSS Statistics 26. **(C)** Dual Y axis with scale X axis of *Zymoseptoria* spp. or *Fusarium* spp. ascospores and value of relative humidity by the 2h timepoints. Values for all the 4 sites were used to generate the positive correlation curve. Graphs with IBM SPSS Statistics 26.

*Zymoseptoria* spp. and *Fusarium* spp. ascospore release data from the microscopy were correlated with weather data to establish any connection between spore release and weather conditions. The 2h spore release data for both ascomycetes were correlated with air temperature (°C), rainfall (mm), relative humidity (%RH), and wind speed (Km/h) using the Spearman’s rho statistical tool (**Table S2**). The air temperature (°C) was negatively correlated to *Zymoseptoria* spp. spore release at all the sites, according to the p – values (> 0.05) and the correlation coefficients (r = - n). Whereas the *Fusarium* spp. ascospore release was only found to negatively correlate with air temperature (°C) at the Lenham site, and no significance was registered for the other sites. Significant (p – value > 0.05) positive correlation coefficients (r = + n) were observed for percentage relative humidity (%RH) with both *Zymoseptoria* spp. and *Fusarium* spp. ascospores across all sites (**Table S2**). For the remaining categories of rainfall (mm) and wind speed (Km/h), the Spearman’s correlations revealed a significant positive correlation between both spore type and rainfall (mm) only at the Carnoustie site. While only at Lenham spore release was found to be significantly, negatively correlated with wind speed (Km/h) data (**Table S2**). The spore abundance over the whole sampling season at 2h intervals for *Zymoseptoria* spp. and *Fusarium* spp. with air temperature (°C) and relative humidity (%RH) are shown on **Figure 4A**. The correlations found between *Zymoseptoria* spp. and *Fusarium* spp. spore release and air temperature (°C), and relative humidity over the four sites plotted by time of the day are shown in **Figures 4B, C**. Negative correlations are indicated when the two lines in the graph bend in opposite directions (e.g., *Zymoseptoria* spp. and air temperature °C). When both lines follow the same curve, the correlation between the two data sets is positive (**Figures 4B, C**, **Table S2**, **Table S3**). These results together suggest that %RH has an impact on the spore release of these two pathogens.

### Metagenomic BLAST taxonomic classification from UK wheat field spore traps

DNA was extracted from the spore trap samples across all sites for the months of June, July, and August followed by WGS. Taxonomic classification was carried out using BLAST followed by phylogenetic analysis (**Figure S1**). The overall taxonomic results from the BLAST metagenomic analysis for the 12 monthly samples are shown as a cladogram tree (**Figure S1**). Most of the BLAST species hits for cellular organisms belonged to the Eukaryote domain, specifically in the Opisthokonta supergroup. Of these, the kingdom Fungi was the most represented in all of the samples. Within the *Ascomycota* phylum, the *Saccharomyceta* clade was present in all samples, with a maximum presence of hits found at Carnoustie in August (80 – 90%). Comparison with the *Basidiomycota* phylum show a division of the classification of reads, into *Agaricomycotina*, *Pucciniomycotina* and *Ustilaginomycotina* with the percentage of hits predominantly in the latter. In the *Pucciniomycotina*, a higher percentage of hits were registered for the Lenham field site in June/August (25–30%), and Swindon in July/August (10–15%). In the *Ustilaginomycotina*, most of the hits (10 – 15%) were identified for Carnoustie in July/August.

### Metagenomic identification of fungal cereal pathogens across UK wheat fields

**Figure 5:**
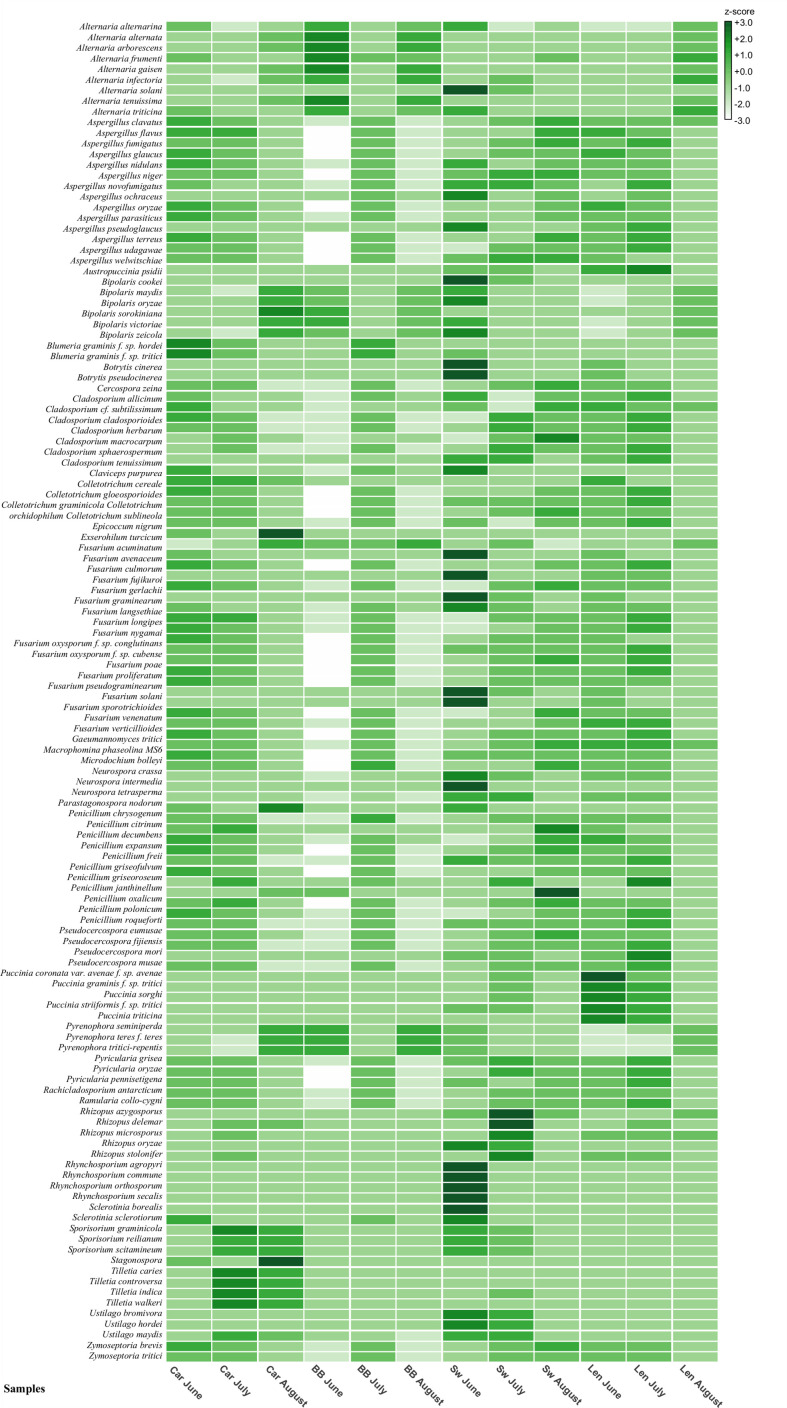
Heat map built in MEGAN 6 summarizing the 150 cereal fungal pathogens species hits recognized by BLAST metagenomic analysis. Species names are on the left-hand side of the map and field site samples names are located on the bottom. Z-scores range: +3/-3. Car, Carnoustie; BB, Bishop Burton; SW, Swindon; Len, Lenham.

Using the BLAST species hits, a total of 150 species (33 different genera) of fungal cereal pathogens were recorded on the spore trap tapes (**Figure 5**; **Figure S2**). Within these, the presence of the six genera previously identified with the microscopy were confirmed (**Figure 5**). A heatmap produced with MEGAN calculated relative abundances between the samples for each species as z-score (-3/+3) (**Figure 5**). The same taxonomic dataset is also shown as a cladogram tree of the species built with MEGAN 6. Which shows the percentage of BLAST hit abundances for each sample in a white (0%) to green (100%) color shade scale (**Figure S2**).

For example, nine species were recognized in the *Alternaria* genus which can infect cereals or impact cereal product quality due to mycotoxin production (**Figure 5**). The highest presence of the *Alternaria* genus was registered at Bishop Burton in June, while the most abundant species amongst all the sites and months was *Alternaria solani* found at Swindon in June (**Figure 5**).

Two *formae speciales* of *Blumeria graminis* were identified: *B. graminis f.* sp. *hordei*, known to be the causal agent of powdery mildew on barley, and *B. graminis f.* sp. *tritici* the causal agent of powdery mildew on wheat. Results from Carnoustie in June and July, and Bishop Burton in July for both *B. graminis f.* sp. shows a higher presence of these pathogens compared to the other months and sites (**Figure 5**).

Hits belonging to the *Cladosporium* genus were divided between seven species that are identified as cereal pathogens. However, from the BLAST analysis the *Rachicladosporium antarcticum* species was recognized as an ubiquitous fungus closely related to plant pathogens belonging to the *Cladosporium* genus (Kang et al., 2019). It was not possible to distinguish the spores of these phylogenetically close species (**Figure S2**) from microscopic analysis. No pattern was observed in the relative abundance between sites or months for these fungi (**Figure 5**).

Within the *Fusarium* genus BLAST species hits, a total of 18 species were recognized as cereal pathogens. The samples from Swindon in June recorded the highest abundance compared to the other samples for the *Fusarium* genus (**Figure 5**). The most abundant species were *F. acuminatum*, *F. culmorum, F. gerlachii, F. graminearum, F. pseudograminearum*, and *F. solani.* BLAST species hits for the *Puccinia* genus, showed the presence of five cereal pathogen species across all sites. The sample from Lenham in June had the highest abundance of *Puccinia* sp., in particular *Puccinia coronata* var. *avenae f.* sp. *avenae* species (**Figure 5**).

Finally, *Z. tritici* relative abundance from the BLAST species hits results appeared to have the same pattern between the sites of Carnoustie and Lenham. A higher abundance of the pathogen was observed in June and July at these sites. In contrast, Swindon had a higher abundance of *Z. tritici* in the months of July and August. At Bishop Burton, the highest *Z. tritici* ascospore abundance was found in July (**Figure 5**).

A larger number of hits were identified that belonged to other fungal species not previously identified from microscopy and classified as major cereal pathogens. These pathogens include: *Aspergillus* spp.*, Botrytis cinerea, Colletotrichum* spp.*, Epicoccum nigrum, Parastagonospora nodorum* (*Septoria nodorum*)*, Penicillium* spp.*, Pyrenophora tritici–repentis, Ramularia collo–cygni, Stagonospora* spp. and *Ustilago maydis* (etc.) (**Figure 5**, **Figure S2**). These species are known to be causal agents of major diseases in cereals and other plants (Sweeney and Dobson, 1998; Freeman et al., 1998; Boudra and Morgavi, 2005; Dean et al., 2012; Różewicz et al., 2021).

### Comparison of metagenomic analysis and microscopy for the identification of fungal cereal pathogens

Fungal species recognized by the BLAST analysis were compared and validated by cross referencing the data with the microscopy analysis, and also using the Kraken2 taxonomic classification tool (Wood et al., 2019) (**Figure S3**; **Table 2**). The methods were compared for the identification of the presence of 20 selected fungal taxa which are cereal pathogens (**Table 2**). For each one of the specific fungal pathogens the presence (+); absence (-); or not – recognized (/) was recorded for each of the methods (Microscopy, BLAST species hits, and Kraken2). All the nine taxa of fungal pathogen within the 20 listed in **Table 2** that were recognized by microscopy were also recognized by the BLAST analysis (*Alternaria* spp.*, Aspergillus* spp.*, Blumeria graminis, Claviceps purpurea, Cladosporium* spp.*, Epicoccum nigrum, Fusarium* spp., *Puccinia* spp.*, Zymoseptoria tritici)*. Only three species out of the nine were recognized by the Kraken2 taxonomy tool (specifically *Aspergillus fumigatus/oryzae, Fusarium graminearum, Zymoseptoria tritici)* (**Table 2**). In contrast, the presence of five fungal taxa (*Botrytis cinerea, Cercospora* spp.*, Colletotrichum* spp.*, Neurospora crassa, Sporisorium* spp.*)* were recognized by both Kraken2 and BLAST analysis, but were not identified by microscopy (**Table 2**). The eight fungal pathogens identified by Kraken2 (*Aspergillus fumigatus/oryzae, Fusarium graminearum, Z. tritici, Botrytis cinerea, Cercospora* spp.*, Colletotrichum* spp.*, Neurospora crassa, Sporisorium* spp.*)* (**Table 2**), were the only fungal hits of the 66 most recognized genera using this method (**Figure S3**). A total of six fungal pathogens, from the 20 selected species, were recognized only using the BLAST analysis (*Parastagonospora nodorum, Pyrenophora teres f. teres, Pyrenophora tritici-repentis, Ramularia collo-cygni, Stagonospora* sp.*, Ustilago maydis)*.

**Table 2:** Recorded presence (+); absence (-); or not – recognized (/) of 20 selected cereal fungal pathogens genera / species from each of the methods of analysis (Microscopy, BLAST, Kraken2).

Cereal Fungal Pathogens	Microscopic Analysis	Blast Analysis	Kraken2 Analysis
*Alternaria* spp.	+	+	–
*Aspergillus* spp. *(fumigatus / oryzae)*	+	+	+
*Blumeria graminis*	+	+	–
*Botrytis cinerea*	/	+	+
*Cercospora* spp.	/	+	+
*Cladosporium* spp.	+	+	–
*Claviceps purpurea*	+	+	–
*Colletotrichum* spp.	/	+	+
*Epicoccum nigrum*	+	+	–
*Fusarium* spp. *(graminearum)*	+	+	+
*Neurospora crassa*	/	+	+
*Parastagonospora nodorum*	/	+	–
*Puccinia* spp.	+	+	–
*Pyrenophora teres f. teres*	/	+	–
*Pyrenophora tritici-repentis*	/	+	–
*Ramularia collo-cygni*	/	+	–
*Sporisorium* spp.	/	+	+
*Stagonospora* sp.	/	+	–
*Ustilago maydis*	/	+	–
*Zymoseptoria* spp.	+	+	+

**Figure 6:**
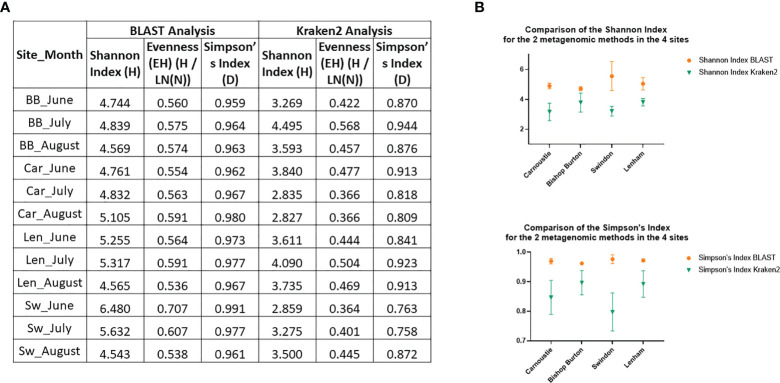
Comparison of the two metagenomic methods, BLAST and Kraken2. **(A)** Table with the monthly values for all the sites of Shannon – Wiener diversity index, its Evenness, and the Simpson’s Index for both methods. Car, Carnoustie; BB, Bishop Burton; SW, Swindon; Len, Lenham; **(B)** XY Plots of variances for the Indexes values between the two methods (BLAST in Orange; Kraken2 in Green) by site.

Comparison between the two metagenomic methods revealed that BLAST analysis is a more efficient method for the recognition of fungal pathogens. The percentages of reads classified from the raw FASTQ reads files from the Kraken2 were between 3% and 8% compared to between 26% and 38% using BLAST, obtained from the same FASTQ reads (**Table S4**). These results were confirmed by the statistical analysis on species diversity indexes. Simpson’s, and Shannon – Wiener indexes were calculated for both methods, and a two – way ANOVA test was used to determine if there was a difference between the methods for each of the diversity indexes. The species diversity for the two metagenomics methods is compared in **Figure 6**. This includes: 1) monthly values, *per* each of the four sites, for the Shannon – Wiener index, the Evenness, and the Simpson’s Index, from the BLAST analysis and from the Kraken2 analysis (**Figure 6A**); 2) analysis of the variances between the two methods, using the monthly samples, for both diversity indexes *per* all the four sites (**Figure 6B**). The diversity indexes related to the BLAST analysis are consistently higher than the variances of the Kraken2 diversity indexes for all the sites (**Figure 6B**), indicating that the BLAST tool can recognize a greater diversity of species than Kraken2. Furthermore, the two – way ANOVA analysis conducted on these data, confirmed that the differences between the two methods are statistically significant with a Column Factor p – value<0.0001 for both, Shannon – Wiener index, and Simpson’s index.

## Discussion

Different types of approaches have been used for the analysis of spore trap samples. The most traditional approach is microscopic analysis and this uses the size, shape and colour to identify and quantify spores. On the other hand, in the last decade molecular based analysis has become more frequent (Jackson and Bayliss, 2011). The various trapping mechanisms and their possible applications in plant biosecurity were reviewed by Jackson and Bayliss (2011), including the advantages and disadvantages of nine types of spore traps and the variety of analyses that can be performed on the samples obtained from each type.

In this work we analyzed air samples from volumetric spore traps, focusing specifically on the presence of cereal fungal pathogen spores present in the air at four UK wheat field sites. To compare the results from each analysis, the same samples were analyzed with both microscopic and metagenomic methods.

Microscopic analysis was performed on spore trap tapes, for the identification and quantification of fungal spores. Approximately 20 morphologically distinct spore types were recognized. Six of these taxa (*Alternaria* spp., *Blumeria graminis, Cladosporium* spp. *Fusarium* spp., *Puccinia* spp., *Zymoseptoria* spp.*)* were frequently found in all samples, making them the most ubiquitous genera present across the sites. The presence of ubiquitous pathogens such as *Cladosporium* spp. and *Alternaria* spp., (D'Amato et al., 1997; Grinn-Gofroń and Rapiejko, 2009) were recorded during the whole season for all the sites. We also used a metagenomic approach where DNA extraction was carried out monthly from June, July, and August, followed by NGS sequencing and classification of BLAST species hits taxonomically (**Figure S1**). For all the four wheat field sites, the majority of the species hits belonged to the fungal kingdom. A total of 33 different genera, which included 150 species of cereal pathogens, were identified by BLAST analysis (**Figure 5**, **Figure S2**). This compares to 63 operational taxonomic units (OTUs) identified from rice fields using DNA metabarcoding of the fungal ITS region (Ortega et al., 2020). Air sampling in the city of Seoul, South Korea identified 80-300 OTUs by sequencing of the fungal ITS region depending on the sampling conditions (dry or wet) and time of the year (Woo et al., 2018). Therefore, the BLAST analysis allows more detailed information on which species are present at each site.

*Blumeria graminis*, was recorded mostly in the first half of the season, which correlates with the life cycle of this pathogen (Dean et al., 2012; Troch et al., 2012). The differences noticeable between the sites could be due to the weather, since *B. graminis* infection occurs in cool and humid weather conditions (Parry, 1990; Dean et al., 2012). This may explain the high presence of *Blumeria graminis* in northern sites where weather conditions are more favorable to its growth. In fact, Carnoustie and Bishop Burton show a higher presence of *Blumeria graminis*, than Lenham and Swindon. These sites are situated in Scotland and the north of England, where weather conditions in summertime are more suitable for this pathogen than in the south of England (**Figure 1**). In this case results from the microscopy analysis, weekly (**Table 1**) and monthly percentages (**Table S1**) for *B. graminis* (both *formae speciales)* agree with the metagenomic results from the BLAST analysis (**Figure 5**) confirming the highest relative abundance at Carnoustie for June and July.

The trend for *Zymoseptoria* spp. ascospores observed using microscopy was the same across all the sites. A high abundance of *Zymoseptoria* spp. ascospores was registered in the first three weeks (May and beginning of June) of sampling followed by a decrease in ascospores in the middle of the sampling season. An increase was then observed in the last week (August) for three of the sites (Carnoustie, Bishop Burton, and Swindon). The production of ascospores from *Z. tritici* comes from sexual reproduction and occurs throughout the growing season (Hunter et al., 1999; Cordo et al., 2017). The *Z. tritici* ascospores are released from stubble present in the field from the previous year. These, ascospores are then carried *via* the wind to land on wheat leaves. Following leaf infection, the pathogen produces asexual pycnidiospores reducing the production of ascospores (Suffert et al., 2019). Sexual reproduction generally increases again at the end of the infection season. This allows the fungus to overwinter, resulting in an increase of ascospore production from August throughout the autumn season (Suffert et al., 2011; Cordo et al., 2017). This life cycle of *Z. tritici* corresponds with the observed airborne ascospore patterns at the four sites. However, the pattern of *Zymoseptoria* spp. ascospore release found from the microscopy analysis was not mirrored from the BLAST analysis for the four sites. For instance, the Lenham and Carnoustie field sites shared a high relative abundance of *Z. tritici* BLAST hits in June and July rather than June and August as suggested by the microscopy. The differences in the results from the two methods, could be due to the uneven distribution of spores on the tapes, which might impact the accuracy of microscopy scoring (Jackson and Bayliss, 2011), this could be further impacted by using a half tape for the analysis.

An example of strong similarity in the results, from both microscopy and metagenomic BLAST analysis, is the high presence of the *Fusarium* genus in the sample from Swindon in June (week 3). *Fusarium* spp. spore counts in week 3 of microscopy were the highest registered in the genus amongst all the samples at 22.47% (**Table 1**) and in terms of the monthly percentage abundances 20.93% (**Table S1**). The same results were also obtained by the metagenomic BLAST analysis (**Figure 5**), which show the highest z-score for five out of 18 *Fusarium* species recognized (*F. acuminatum; F. culmorum; F. graminearum; F. pseudograminearum; F. solani*), supporting that the sample from Swindon June has the highest presence of *Fusarium* spp. Compared to other samples. Generally, the spores belonging to the *Fusarium* species, are known to be present in the air throughout the whole season (Karlsson et al., 2021).

Volumetric spore traps used in this experiment allowed data to be obtained at two-hour time points (2 h) which was compared to weather data to understand if any correlation between spore release and climate conditions was present. Weather conditions such as humidity can influence the infectivity rate of *Z. tritici* (Fones et al., 2017). Air humidity and air velocity are known as factors which can influence spore release in the atmosphere (Pasanen et al., 1991). Comparison of *Zymoseptoria* spp. and *Fusarium* spp. Ascospore types with relative humidity (%RH) performed in this study, showed a consistent and strong positive correlation, these results were identical for all 4 sites. In contrast, air temperature (°C) negatively affects *Zymoseptoria* spp. spore release, the data from the 4 sites show a significant negative correlation for the Spearman’s rank (**Table S2**). *Z. tritici* ascospore release in Argentina was previously found to be positively corelated with %RH and negatively correlated with temperature which is in agreement with our findings (Cordo et al., 2017). Trail et al., 2002 also found *F. graminearum* ascospore discharge to increase at %RH greater than 92% under light conditions (TrailKeller et al., 2014). Previous studies found the optimum temperature for *F. graminearum* ascospore release was at 16.6°C (Tschanz et al., 1976). In contrast, our results identified a negative correlation with temperature only at Lenham, which was the most Southern UK site. Therefore, microscopy can be useful to understand the relative concentration of fungal pathogens in the air and to identify external factors that impact spore release. This information may be used to inform the timing and application of fungicides for disease control.

Overall, microscopy allows the presence and concentration of specific airborne pathogens present in the area to be recorded. However, microscopic analysis is time consuming and requires trained personnel able to recognize a broad range of genera or species. Furthermore, the microscopy from spore trap tapes can be affected by a variety of factors including types of adhesive. These can influence the microscope focus plane where the spores appear, or their ability to adhere in one/multiple layers or aggregate due to humidity and water drops (Jackson and Bayliss, 2011). Microscopic analysis rely exclusively on manual counting of spores, in which error can vary depending on the counting technique used by the operator (Comtois et al., 1999; Sterling et al., 1999). No method is completely reliable unless the whole slide is counted (Comtois et al., 1999). In this work, the 12 transverses (representative of the 2 h sampling) (Lacey and West, 2007) gave the closest results to when the whole slide is counted with small error in comparison to other counting methods, giving a better estimation of spore presence in a shorter time.

In order to evaluate the efficiency of the BLAST analysis, a second taxonomy tool was employed. The same samples FASTQ reads were run with the Kraken2 software, which was included in the OmicsBox package. The two metagenomic methods were inconsistent with each other for fungal species recognition. In fact, Kraken2 recognized only some of the fungal species present in the metagenome, within the top 66 genera recognized only eight were fungal pathogens (**Figure S3**; **Table 2**). The two methods have a difference in efficiency demonstrated by the percentage of the total reads classified (**Table S4**). Percentages of reads classified by the BLAST analysis are between three and 12 times greater than the reads classified by Kraken2. Another significant difference between the two methods can be observed from the two Diversity indexes, Shannon – Wiener, and Simpson’s (**Figure 6**). A major difference in the cereal fungal pathogens’ presence registered from the Microscopy, BLAST and Kraken2 analysis is shown in **Table 2**. Which displays spores from 20 major cereal fungal pathogens as; not recognized (/); presence (+); absence (-). This demonstrates that pathogens such as *Alternaria* spp., *Puccinia* spp.*, Blumeria graminis, Cladosporium* spp., and *Claviceps purpurea*, are not found in the Kraken2 database, but are recognized by both Microscopy and BLAST analysis (**Table 2**). Only three pathogens out of the 20 listed in **Table 2** are recognized by all three methods, these are *Aspergillus* spp. *(fumigatus/oryzae), Fusarium* spp. *(graminearum)*, and *Zymoseptoria* spp. *(tritici)*. BLAST taxonomic analysis registered the presence for all of the 20 selected cereal pathogens, these results are for the most part supported by either the microscopy results or/and the Kraken2 analysis. Similar results were recently published by Jo et al. (2020) where the authors use both, Kraken2 and a BLASTx metagenomic analysis for peach. The authors come to similar conclusions that the Kraken2 database for *Fungi* is not as extensive as the NCBI database used in the BLAST analysis (Jo et al., 2020).

Various metagenomic approaches can be applied to spore trap tapes, some of which can deliver different types of information. For instance, the method applied in this study focused on presence/absence of specific cereal pathogens in the field. Methods such as NGS with subsequent BLAST taxonomic classification may have implications for identification of pathogens important for plant biosecurity (Jackson and Bayliss, 2011). With which, the presence of specific Quarantine Pests could be easily and quickly identified. While recognizing the presence of specific species with methods such as microscopic analysis is difficult and time consuming. Moreover, alternative methods that use metagenomic solutions can be used to determine the quantity and type of the pathogens in the field (Aguayo et al., 2018; Woo et al., 2018). This can be performed through quantitative PCR evaluating the gene copy number (Woo et al., 2018) or with metabarcoding (Aguayo et al., 2018). It would be interesting in future studies to compare metagenomics and metabarcoding using ITS sequencing of the spore trap tapes to determine if metabarcoding is more sensitive since it is also cheaper than the WGS, used here. These approaches can substitute the traditional, time consuming, microscopy approach.

Microscopy gives detailed information on abundance and timing of fungal spores present. In contrast, the metagenomic approach coupled with BLAST analysis provides a more rapid, accurate recognition and estimate of abundances of the fungal cereal pathogens. Results from the Kraken2 taxonomic classification were instead less accurate and many species were not present in the software database and therefore not recognized.

Future approaches using metagenomics may be useful for the identification of crop pathogens and prediction of disease outbreaks without bias or knowledge of which pathogens are present. For example, 150 cereal pathogens were detected here using metagenomic BLAST analysis. The future development of the method to correlate the abundance of pathogens identified by metagenomic BLAST analysis to weather data could possibly be used to inform the timing and application of fungicides for disease control.

## Data availability statement

The datasets presented in this study can be found in online repositories. The names of the repository/repositories and accession number(s) can be found below: https://www.ncbi.nlm.nih.gov/, PRJNA872486.

## Author contributions

PP and AF designed the experiment. PP and CL carried out sampling and microscopic and metagenomic experiments. AT contributed to the sampling and microscopic analysis. PP carried out the analysis of the results. PP and AF wrote the manuscript. SK, JB, and AF revised the manuscript. All authors contributed to the article and approved the submitted version.

## Funding

This work was supported by the SFI Strategic Partnerships Programme (16/SPP/3296) and SFI Career Development Award (15/CDA/3451).

## Acknowledgments

The authors want to thank Agrii plc and Origin enterprises plc for providing access to the UK field sites. Special thanks to Francesca Salinari and David Langton for the help with the sample collection.

## Conflict of interest

The authors declare that the research was conducted in the absence of any commercial or financial relationships that could be construed as a potential conflict of interest.

## Publisher’s note

All claims expressed in this article are solely those of the authors and do not necessarily represent those of their affiliated organizations, or those of the publisher, the editors and the reviewers. Any product that may be evaluated in this article, or claim that may be made by its manufacturer, is not guaranteed or endorsed by the publisher.

## Supplementary material

Supplementary Figure 1 Cladogram tree of BLAST species hits distribution; each node contains 12 boxes representing the 12 samples (3 monthly samples for the four UK wheat field sites). Color shading of the boxes indicates the percentage of BLAST hits relative abundance in that specific clade for the specific sample. The white colour represents 0% increasing through green shading to dark green representative of 100%. Tree built with MEGAN 6.

Supplementary Figure 2 BLAST species hits for 150 cereal fungal pathogen species, distributed in a cladogram tree; each node contains 12 boxes representing the 12 samples (3 monthly samples for the four UK wheat field sites). Colour shading of the boxes indicates the percentage of BLAST hits relative abundance in that specific clade for the specific sample. The white color represents 0% increasing through green shading to dark green representative of 100%. Tree built with MEGAN 6.

Supplementary Figure 3 Stacked taxa bar chart showing the relative abundance at the general level from the Kraken2 tool, only eight out of the most abundant 66 genera recognized were fungal organisms. Bar chart was built using OmicsBox (BioBam).
