# Supplementary material for: Comparison of microscopic and metagenomic approaches to identify cereal pathogens and track fungal spore release in the field
Source: Front Plant Sci. 2022 Oct 20;13:1039090. doi: 10.3389/fpls.2022.1039090 (PMC9630935; doi:10.3389/fpls.2022.1039090)

Supplementary Table 1

Monthly percentages of fungal pathogens based on the averages from the 2 weeks data point, for the four field sites

**Field Site**	Carnoustie				Bishop Burton				Swindon				Lenham			
Species \ Month	May*	June	July	August	May*	June	July**	August	May*	June	July	August	May*	June	July	August**
Alternaria spp.	0.58%	3.20%	1.02%	2.22%	1.19%	1.44%	6.28%	4.04%	1.29%	1.20%	1.76%	1.46%	1.14%	2.45%	2.14%	5.26%
Blumeria graminis	13.20%	7.27%	4.80%	0.07%	3.99%	4.03%	0.80%	0.03%	1.01%	0.25%	0.57%	0.06%	0.85%	0.47%	0.99%	1.10%
Cladosporium spp.	42.49%	66.69%	90.72%	31.49%	16.58%	56.88%	89.05%	46.80%	11.19%	13.38%	44.60%	83.50%	19.82%	48.22%	90.07%	76.75%
Fusarium spp.	6.66%	2.70%	0.58%	0.95%	9.63%	3.55%	0.12%	0.57%	14.21%	20.93%	0.68%	2.58%	10.21%	2.78%	1.74%	4.39%
Puccinia spp.	8.28%	7.84%	2.01%	0.30%	1.22%	2.81%	3.53%	0.49%	3.27%	3.98%	1.74%	0.43%	5.80%	8.22%	3.15%	9.35%
Zymoseptoria spp	12.22%	2.81%	0.34%	11.63%	10.90%	3.74%	0.06%	2.91%	17.70%	13.68%	0.11%	3.97%	13.91%	11.92%	0.79%	2.55%
Other	16.57%	9.48%	0.54%	53.35%	56.49%	27.55%	0.15%	45.17%	51.32%	46.58%	50.54%	8.02%	48.27%	25.95%	1.11%	0.59%

Supplementary Table 2

Statistical results of the nonparametric Spearman's rank correlation coefficient for *Z. spp* and *F. spp* spore release, with weather data

Zymoseptoria tritici spore release	Spearman's rho	Air Temperature (°C)	Rainfall (mm)	Relative Humidity (%RH)	Wind Speed (Km/h)
**Carnoustie**	Correlation Coefficient	-.165	.113	.260	-.002
	Sig. (2-tailed)	0.000018	0.003556	9.1833E-12	.953
	N	668	668	668	668
**Bishop Burton**	Correlation Coefficient	-.108	.015	.218	-.045
	Sig. (2-tailed)	.009	.724	9.7109E-8	.280
	N	588	588	588	588
**Swindon**	Correlation Coefficient	-.098	.013	.089	-.054
	Sig. (2-tailed)	.011	.738	.022	.163
	N	670	670	670	670
**Lenham**	Correlation Coefficient	-.340	-.063	.237	-.082
	Sig. (2-tailed)	2.1354E-17	.128	6.4238E-9	.048
	N	588	588	588	588

Fusarium spp. spore release	Spearman's rho	Air Temperature (°C)	Rainfall (mm)	Relative Humidity (%RH)	Wind Speed (Km/h)
**Carnoustie**	Correlation Coefficient	-0.70	.150	.149	.044
	Sig. (2-tailed)	.071	0.000099	0.000117	.259
	N	668	668	668	668
**Bishop Burton**	Correlation Coefficient	-.040	.048	.245	.006
	Sig. (2-tailed)	.339	.247	1.8786E-9	.893
	N	588	588	588	588
**Swindon**	Correlation Coefficient	-.067	.026	.124	.077
	Sig. (2-tailed)	.082	.494	.001	.045
	N	669	669	669	669
**Lenham**	Correlation Coefficient	-.221	-.049	.180	-.086
	Sig. (2-tailed)	6.0613E-8	.238	0.000011	.036
	N	588	588	588	588

Supplementary Table S3.

Summary of the spearman's correlation between relative ascospore release of *Zymoseptoria* spp. / *Fusarium* spp. and weather data over the 4 UK sites. (-) negative correlation; (+) postive correlation; (0) no correlation

Zymoseptoria spp.					*Fusarium* spp.				
**Site**	**air temperature**	**rainfall**	**relative humidity**	**wind**	**Site**	**air temperature**	**rainfall**	**relative humidity**	**wind**
**Carnoustie**	-	+	+	0	**Carnoustie**	0	+	+	0
**Bishop Burton**	-	0	+	0	**Bishop Burton**	0	0	+	0
**Swindon**	-	0	+	0	**Swindon**	0	0	+	+
**Lenham**	-	-	+	-	**Lenham**	-	0	+	-

Supplementary Table 4

Comparison of classified reads / proteins percentages between the two metagenomic methods

Site_month	Reads FASTQ	BLAST			Kraken2	
		Tot protein prediction	Tot protein BLAST	% proteins classified	Reads Classified	% Reads Classified
Car_June	7798087	363273	128845	35.47%	301701	3.87%
Car_July	6860526	548306	173889	31.71%	260513	3.80%
Car_August	8908787	716083	230085	32.13%	380551	4.27%
BB_June	6300545	242287	85138	35.14%	186070	2.95%
BB_July	4405590	393767	135646	34.45%	186253	4.23%
BB_August	6569272	537188	177437	33.03%	261551	3.98%
Sw_June	9740785	136580	52169	38.20%	263774	2.71%
Sw_July	6235410	301166	78340	26.01%	498113	7.99%
Sw_August	5961988	421562	129664	30.76%	237112	3.98%
Len_June	10414256	402856	140638	34.91%	300705	2.89%
Len_July	5826176	204831	60437	29.51%	250923	4.31%
Len_August	7962778	596549	178631	29.94%	306464	3.85%

Supplementary Figure 1

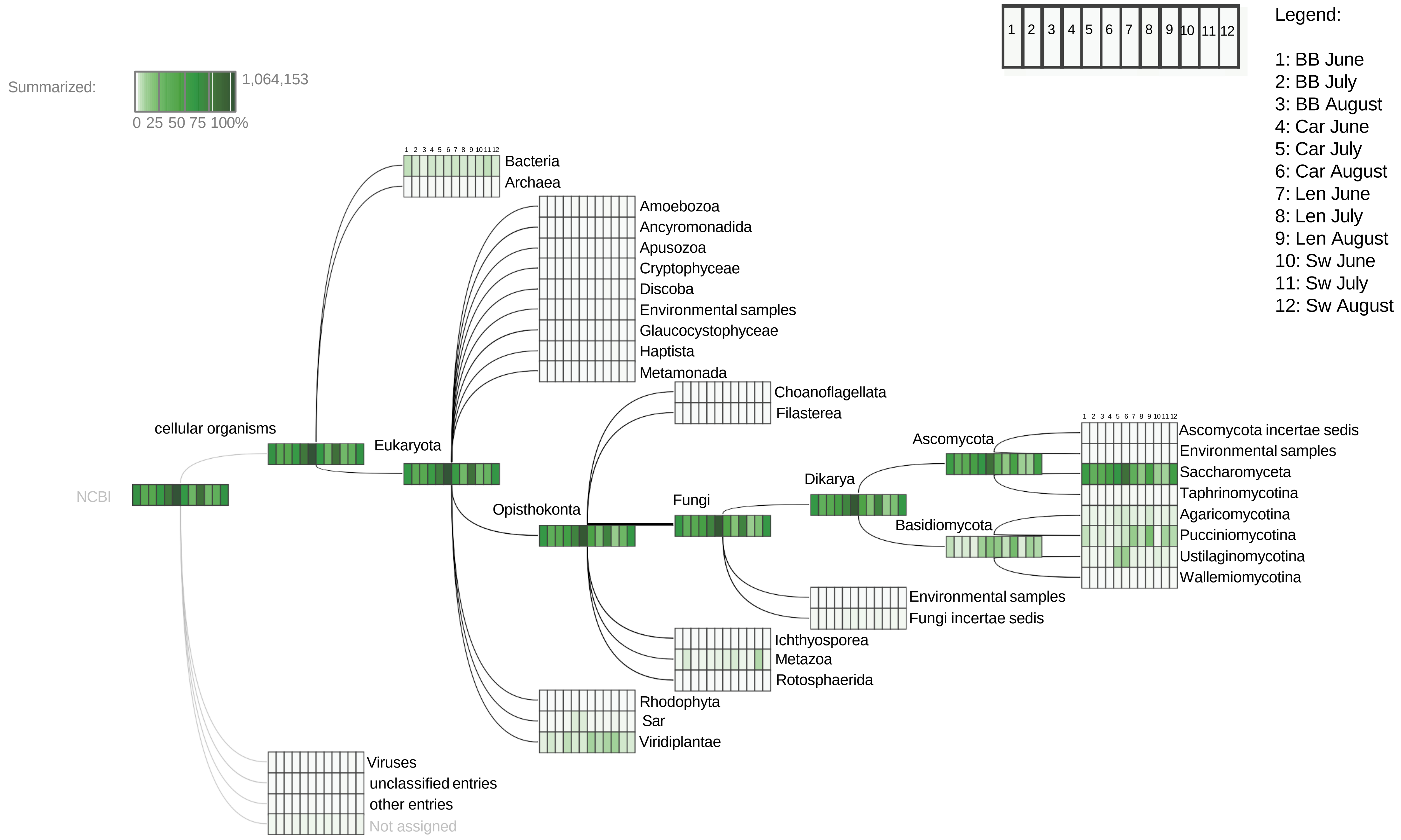

Supplementary Figure 2

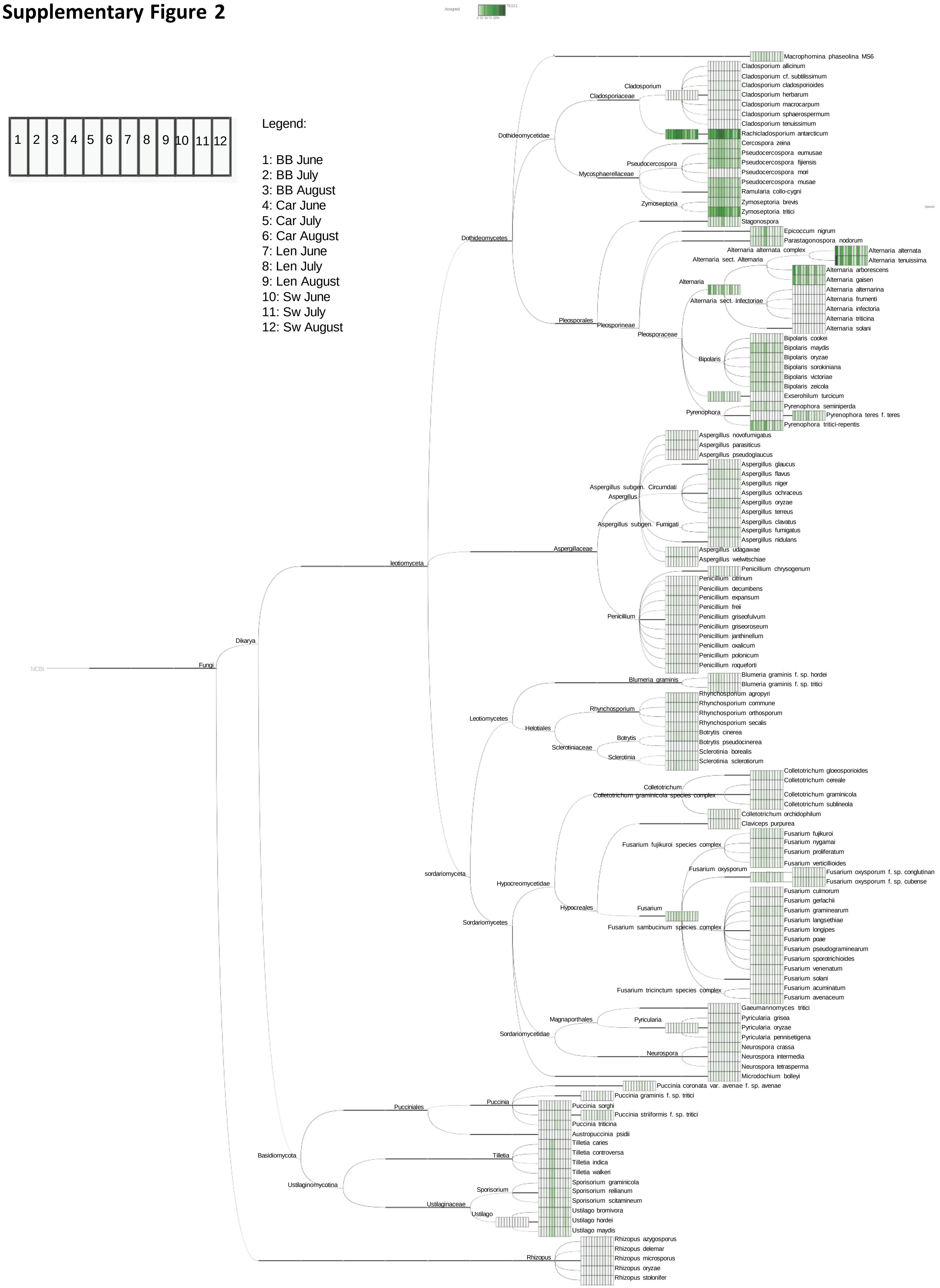

Supplementary Figure 3

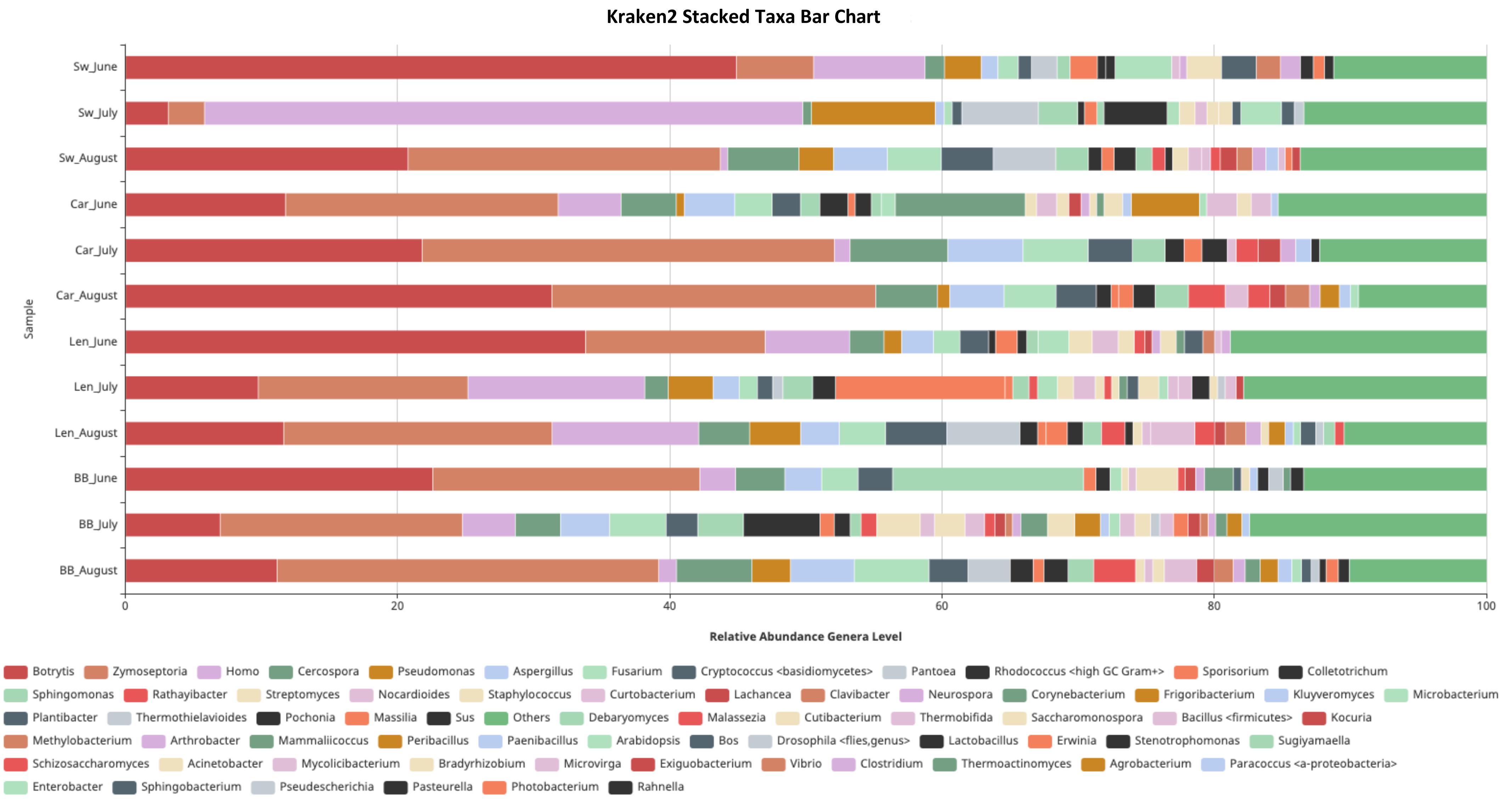

Supplement: Supplementary Figure 1 — Cladogram tree of BLAST species hits distribution; each node contains 12 boxes representing the 12 samples (3 monthly samples for the four UK wheat field sites). Color shading of the boxes indicates the percentage of BLAST hits relative abundance in that specific clade for the specific sample. The white colour represents 0% increasing through green shading to dark green representative of 100%. Tree built with MEGAN 6. [file DataSheet_1.pdf]
